# Concurrent HDAC and mTORC1 Inhibition Attenuate Androgen Receptor and Hypoxia Signaling Associated with Alterations in MicroRNA Expression

**DOI:** 10.1371/journal.pone.0027178

**Published:** 2011-11-07

**Authors:** Leigh Ellis, Kristin Lehet, Swathi Ramakrishnan, Remi Adelaiye, Kiersten M. Miles, Dan Wang, Song Liu, Peter Atadja, Michael A. Carducci, Roberto Pili

**Affiliations:** 1 Roswell Park Cancer Institute, Genitourinary Program, Grace Cancer Drug Center, Buffalo, New York, United States of America; 2 Roswell Park Cancer Institute, Department of Bioinformatics, Buffalo, New York, United States of America; 3 Novartis Biomedical Research Institute, Shanghai, China; 4 The Sidney Kimmel Comprehensive Cancer Center at Johns Hopkins, Baltimore, Maryland, United States of America; Technische Universität München, Germany

## Abstract

Specific inhibitors towards Histone Deacetylases (HDACs) and Mammalian Target of Rapamycin Complex 1 (mTORC1) have been developed and demonstrate potential as treatments for patients with advanced and/or metastatic and castrate resistant prostate cancer (PCa). Further, deregulation of HDAC expression and mTORC1 activity are documented in PCa and provide rational targets to create new therapeutic strategies to treat PCa. Here we report the use of the c-Myc adenocarcinoma cell line from the *c-Myc* transgenic mouse with prostate cancer to evaluate the *in vitro* and *in vivo* anti-tumor activity of the combination of the HDAC inhibitor panobinostat with the mTORC1 inhibitor everolimus. Panobinostat/everolimus combination treatment resulted in significantly greater antitumor activity in mice bearing androgen sensitive Myc-CaP and castrate resistant Myc-CaP tumors compared to single treatments. We identified that panobinostat/everolimus combination resulted in enhanced anti-tumor activity mediated by decreased tumor growth concurrent with augmentation of p21 and p27 expression and the attenuation of angiogenesis and tumor proliferation via androgen receptor, c-Myc and HIF-1α signaling. Also, we observed altered expression of microRNAs associated with these three transcription factors. Overall, our results demonstrate that low dose concurrent panobinostat/everolimus combination therapy is well tolerated and results in greater anti-tumor activity compared to single treatments in tumor bearing immuno-competent mice. Finally, our results suggest that response of selected miRs could be utilized to monitor panobinostat/everolimus *in vivo* activity.

## Introduction

Treatment for advanced prostate cancer currently involves hormone therapies that lower serum testosterone and antagonize the transcriptional capabilities of the androgen receptor (AR) by targeting its ligand binding domain. Initially effective, these therapies are eventually ‘adapted’ to, enabling the cancer to survive in a low androgen environment. This results in the development of a lethal PCa phenotype, castrate-resistant prostate cancer (CRPC). Currently, therapies including the microtubule inhibitors docetaxel and cabazitaxel, and the recently approved abiraterone and the autologous immunotherapy sipuleucel T are available therapies to patients with CRPC. Although these therapies are life prolonging, additional treatment options are still required.

Targeted therapies have emerged as promising agents for novel therapeutic interventions in PCa. Thereby understanding specific genetic and/or epigenetic alterations we can better strategize how to utilize targeted therapies to their fullest potential. PCa can be characterized by four predominant genetic and cellular modifications which include the presence of the *TMPRSS2-ERG* gene fusion [1]; loss of phosphatase and tensin homolog *(Pten)* tumor suppressor function ultimately resulting in constitutive PI3K-pathway activation [2]; amplification of the oncogene *Myc* [3]; and the amplification, over-expression or mutation of the AR [4], [5]. More recently epigenetic changes including deregulation of small non-coding RNAs called microRNA as well as histone deacetylases (HDACs) have been documented in PCa pre-clinical and clinical studies [6], [7].

A primary target of the PI3K-pathway is Akt and its downstream effector mammalian target of rapamycin (mTOR) [8]. mTOR promotes cellular protein synthesis and is highly involved in cell cycle progression, proliferation, apoptosis, autophagy and angiogenesis [9]. mTOR signaling is organized into two main mutiprotein complexes; mTORC1 (mTOR complex 1) and mTOR2 (mTOR complex 2). mTORC1 is the molecular target of the FDA approved mTOR inhibitor rapamycin and its analogs everolimus and temsirolimus which act to antagonize mTORC1 activity via allosteric inhibition [10], [11].

HDACs are documented to play a major role in the progression of PCa [6], [12]. While HDACs are an important component of transcriptional co-repressor complexes mediating gene transcription via deacetylation of histones, they also regulate the activity of non-histone proteins including two critical transcription factors in PCa, HIF-1α [13] and AR [14] via deacetylation. The HDAC inhibitors romidepsin and vorinostat, have been approved to treat cutaneous T cell lymphomas. While mTORC1 [15] and HDAC [12] inhibitors show great promise as monotherapies, it maybe in combination strategies where these agents reach their fullest clinical potential. For that reason, multiple clinical trials are currently pursuing optimum combination strategies to best utilize these targeted therapies in multiple cancer types, including PCa.

Within, we utilize the mouse prostate cancer cell line Myc-CaP generated from the Hi-Myc murine model of PCa [16], [17] which drives the expression of human c-Myc by the androgen receptor dependent rat probasin promoter to demonstrate that low dose combination of the HDAC inhibitor panobinostat and the mTORC1 inhibitor everolimus *in vitro* and *in vivo* result in greater anti-tumor activity than single agent treatment in a murine model of PCa. Overall panobinostat/everolimus combination resulted in a significant reduction in angiogenesis and tumor cell proliferation when compared to single agent treatments. These combination effects were associated with induction of the cyclin dependent kinase inhibitors p21 and p27. Significant loss of transcriptional activity driven by HIF-1α, c-Myc and AR was also observed. Further, we demonstrate a distinct regulation of two oncogenic miRs associated with PCa and HIF-1α, c-Myc and AR signaling. These miRs could be utilized to monitor response to therapy. The cooperative effect from combination therapy on important signaling pathways likely explains the greater therapeutic effect *in vivo*.

## Results

### Myc-CaP cell line *in vitro* sensitivity to panobinostat and everolimus

**Figure 1:**
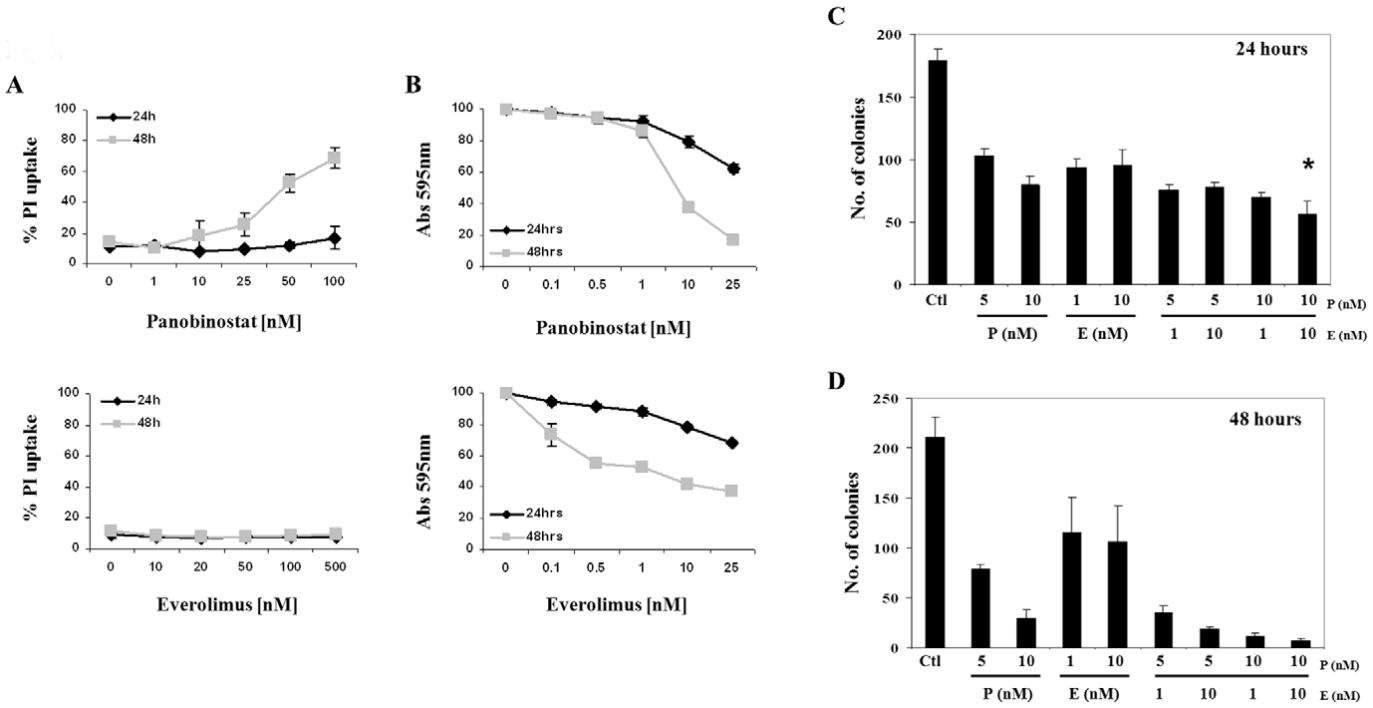
Myc-CaP sensitivity to HDAC and mTORC1 inhibition. (A) Myc-CaP cell lines were incubated with indicated concentrations of panobinostat or everolimus for 24 and 48 hours. Cell viability was determined by the uptake of PI and FACS analysis. (B) Myc-CaP cells were incubated with indicated concentrations of panobinostat or everolimus for 24 and 48 hours. Cell were fixed and stained with 10% MeOH in crystal violet. Final cell growth was determined by quantitating the absorbance at an optical density of 570 nm. (C–D) The clonogenic potential of Myc-CaP cell lines treated for 24 and 48 hours with panobinostat or everolimus alone or in combination and quantitated by image J analysis. Results shown represent the mean ± SE of 3 separate experiments. * p<0.05.

Myc-CaP cell lines cultured *ex vivo* were exposed to increasing concentrations of panobinostat and everolimus for 24 and 48 hours and cell membrane permeability was assessed by uptake of propidium iodide (PI). As shown in Figure 1A (top and bottom panel), Myc-CaP cells were sensitive to the cytotoxic effects of panobinostat in a dose and time dependent manner. Conversely, increasing concentrations of everolimus did not display any cytotoxic effects towards Myc-CaP cells. Because Myc-CaP cell lines remained resistant to the cytotoxic effects of everolimus it was hypothesized that Myc-CaP cells would be sensitive to everolimus growth inhibitory effects. Myc-CaP cells treated with non-cytotoxic concentrations of panobinostat and everolimus for 24 and 48 hours were assessed for cell growth by colorimetric absorbance of Myc-CaP cells fixed and stained with 10% MeOH in crystal violet. Figure 1B (top and bottom panel) shows that Myc-CaP cells were sensitive to growth inhibitory effects induced by panobinostat and everolimus in a time and dose dependent manner. From figure 1A and B we chose to explore clonogenic survival assays with non-cytotoxic concentrations of panobinostat and everolimus to evaluate the long term effects of panobinostat and everolimus as single or combination treatments. Non-cytotoxic concentrations were based on concentrations of either compound that did not induce loss of cell viability but induced decrease in cell growth. Figure 1C and D demonstrates quantitation of colony growth. These results indicate that low non-cytotoxic concentrations of panobinostat (10 nM) and everolimus (10 nM) in combination have significant inhibition of clonogenic survival over single treatments at 24 hours. Based on our clonogenic data, concentrations of panobinostat (10 nM) and everolimus (10 nM) were chosen for further *in vitro* analyses.

### Non-cytotoxic concentrations of panobinostat/everolimus combination induce cell cycle arrest and not apoptosis

**Figure 2:**
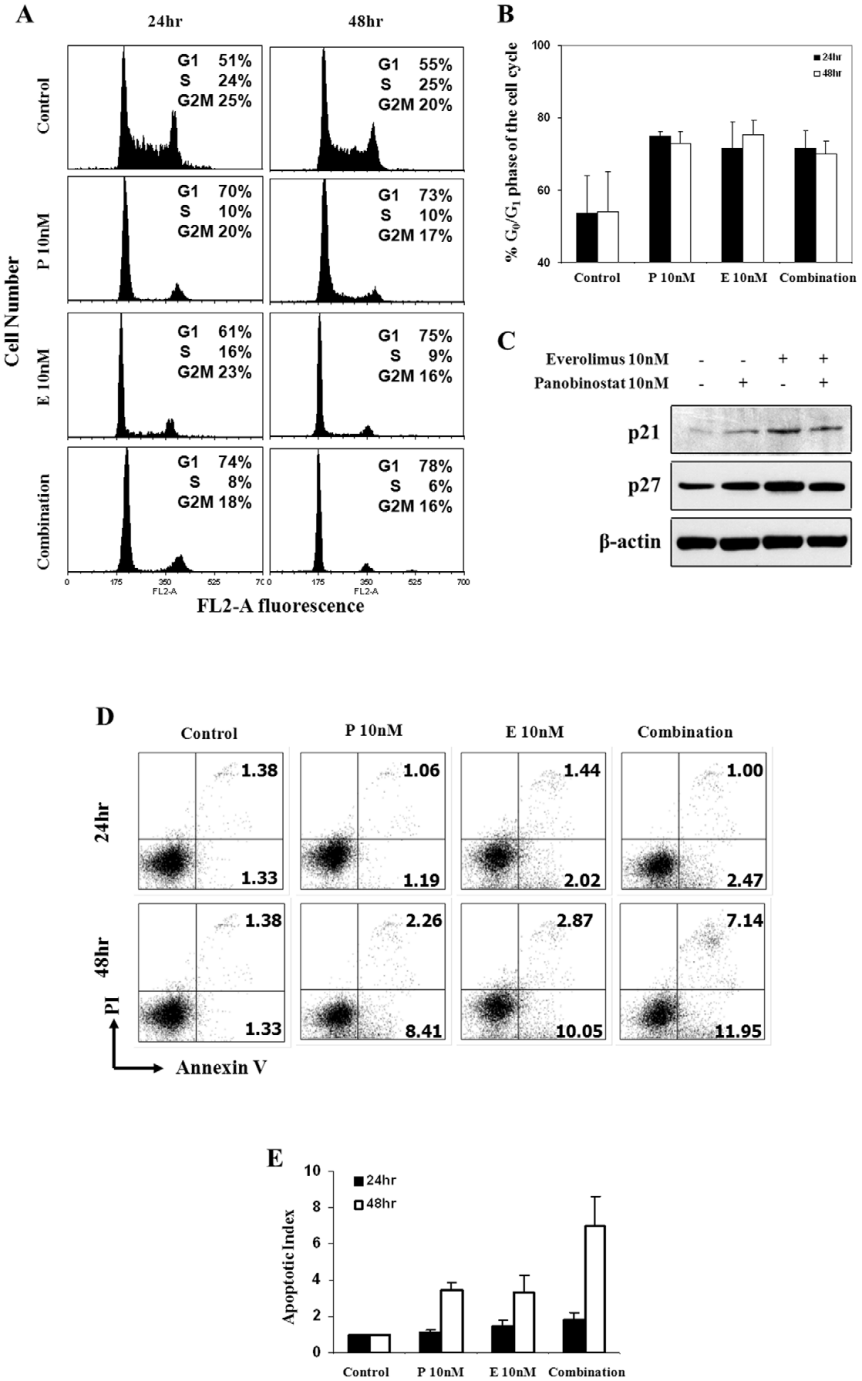
HDAC and mTORC1 inhibition induce cell cycle arrest but not apoptosis. (A) Myc-CaP cells (4×10^5^) treated with indicated concentrations of panobinostat [P] and everolimus [E] alone or in combination for 24 and 48 hours. Cell cycle analysis was performed by FACS after staining fixed/permeabilized cells with PI. Cell cycle distribution of cells in G_0_/G_1_, S, G_2_/M and SubG_1_ (<2 N DNA content) are indicated. (B) Myc-CaP cell lines treated with indicated concentrations of panobinostat or everolimus alone or in combination and quantitation G_0_/G_1_ cells was calculated. (C) Western blot analysis of p21 and p27 proteins. MycCaP cells were treated with 10 nM everolimus, 10 nM panobinostat or combination for 24 hours. Cell lysates were then subjected to immunoblot incubated with the respective antibodies. β-actin served as loading control. (D) FACS scatter plots displaying Myc-CaP cells stained with PI and annexin-V after treatment with 10 nM everolimus, 10 nM panobinostat or combination for 24 and 48 hours. (E) Quantitation of (D) indicates that combination treatment results in the greater increase of the apoptotic index. Results shown in B and E represent the mean ± SE of 3 separate experiments.

Because low dose concentrations of panobinostat and everolimus in combination resulted in greater loss of clonogenic survival it was our objective to determine if this was due to inhibition of cell cycle progression or induction of apoptosis. Treatment of Myc-CaP cells with 10 nM panobinostat and 10 nM everolimus individually or in combination for 24 and 48 hours indicates that both single and combination treatments did not induce cell death as no accumulation of cells in SubG_1_ were observed. Inhibition of cell cycle progression was induced, evident by a loss of S phase and a concomitant increase in the G_0_/G_1_ phase (Fig. 2A and B). Western blot analysis reveals that after 24 h of treatment with panobinostat we see a modest induction of both p21 and p27 while everolimus induced a stronger response of both cdk inhibitors. Panobinostat/everolimus combination did not result in increased protein expression of p21 or p27 (Fig. 2C). Further confirmation that induction of apoptosis was not significantly increased by single or combination treatments over 24 and 48 hours is indicated by staining of treated and untreated Myc-CaP cells with annexin-V and PI which demonstrates that only small populations of cells stain positive for these apoptotic markers with combination treatment resulting in an enhanced but not significant increase as compared to untreated and single treated Myc-CaP cells (Fig. 2D and E).

### Panobinostat/everolimus combination results in reduced tumor burden in mice bearing androgen sensitive or castrate resistant Myc-CaP tumors

**Figure 3:**
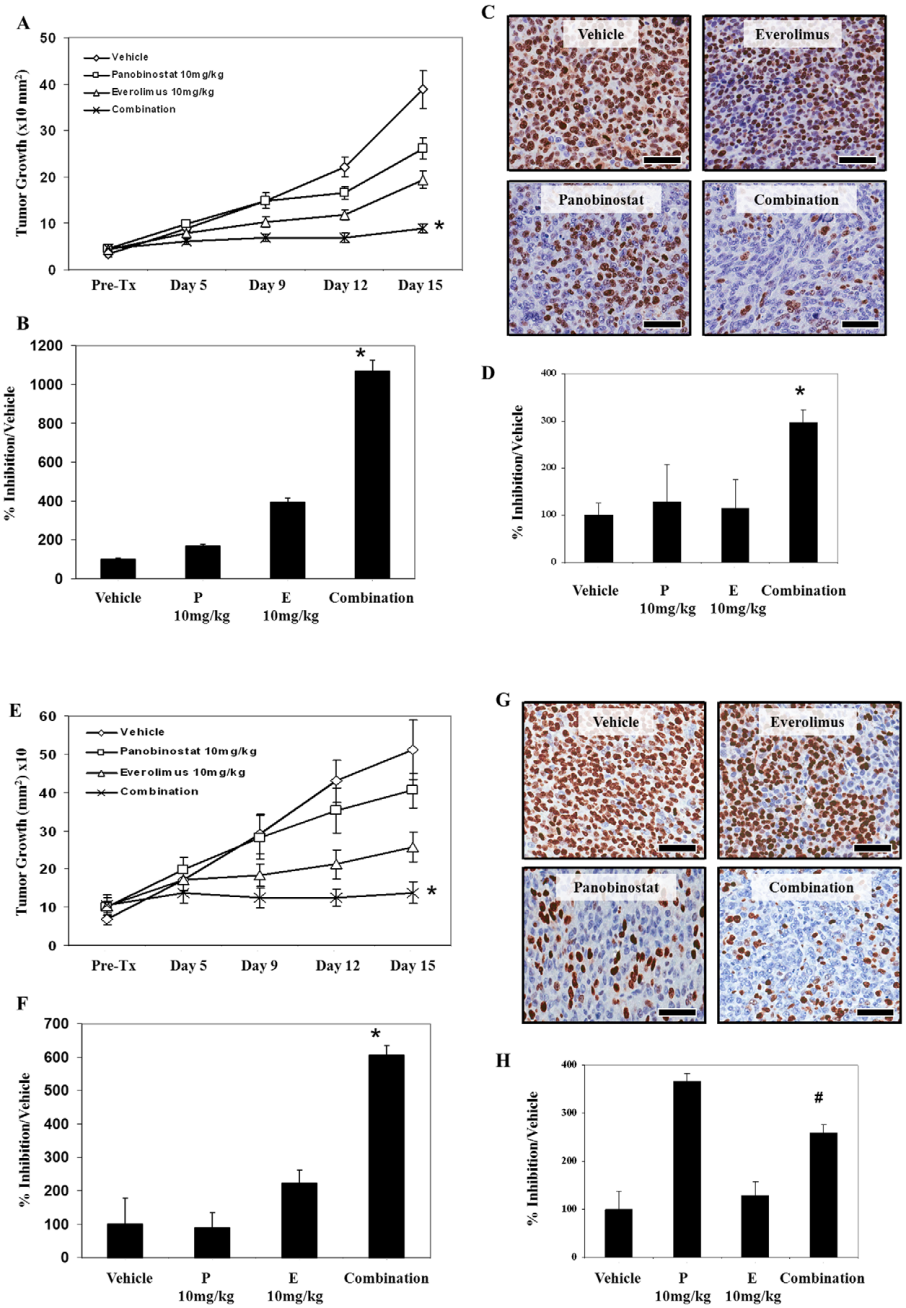
Concurrent HDAC and mTORC1 inhibition mediate increased anti-tumor activity and with loss of tumor proliferation in mice bearing Myc-CaP tumors. (A) Myc-CaP/AS tumors (∼5 mm^2^) were transplanted into the flank of wild type intact FVB intact male mice. Approximately ten (10) days post transplantation mice were treated daily with 10 mg/kg everolimus [E] orally, 10 mg/kg panobinostat [P] IP or both agents concurrently for a total of 15 days. Tumors were measured twice weekly. Mean ± SE, *n*≥10. (B) Excised tumors were weighed to assess the therapeutic efficacy of each treatment schedule. Mean ± SE, *n*≥7. (C–D) Tumor samples from A (*n* = 3) were collected and fixed in formalin for IHC analysis. Four micron (4 µM) sections were stained for the cell proliferation marker Ki67. Magnification (×40); Scale bar = 50 µM. (E) Myc-CaP/CR tumors (∼5 mm^2^) were transplanted into the flank of wild type castrated FVB male mice. Mice were treated daily with 10 mg/kg everolimus orally, 10 mg/kg panobinostat IP or both agents concurrently for a total of 15 days. Tumors were measured twice weekly. Mean ± SE, *n*≥10. (F) Excised tumors were weighed to assess the therapeutic efficacy of each treatment schedule. Mean ± SE, *n*≥7. (G–H) Tumor samples from A (*n* = 3) were collected and fixed in formalin for IHC analysis. Four micron (4 µM) sections were stained for the cell proliferation marker Ki67. Magnification (×40); Scale bar = 50 µM. * indicates that combination treatment is significant compared to either single agent treatment. *P*<0.05. # Note in figure 3H, combination was significant when compared to everolimus treatment (p<0.05) but not to panobinostat treatment.

To further investigate the therapeutic potential of panobinostat/everolimus combination for the treatment of prostate cancer, pre-clinical therapy studies were conducted. Myc-CaP/AS (androgen sensitive) or Myc-CaP/CR (castrate resistant) tumor pieces (∼5 mm^2^) were transplanted unilateral to intact or castrated male FVB mice respectively. Tumor bearing animals were then treated with 10 mg/kg panobinostat (ip), 10 mg/kg everolimus (oral gavage), or the combination for 15 days on a QD ×7 schedule. Treatment with panobinostat alone resulted in a modest decrease in mean tumor proliferation and volume in androgen sensitive (Fig. 3A–D) and castrate resistant Myc-CaP tumors (Fig. 3E and F). Interestingly, panobinostat single treatment mediated a strong reduction in tumor proliferation as indicated by IHC staining for Ki67 (Fig. 3G and H) compared to vehicle treated controls. Everolimus also induced a modest decrease in tumor growth, size and proliferation of androgen sensitive (Fig. 3A–D) and castrate resistant Myc-CaP tumors (Fig. 3E–H), while panobinostat/everolimus combination therapy significantly reduced tumor proliferation and volume in both Myc-CaP/AS (Fig. 3A–D) and Myc-CaP/CR (Fig. 3E–H) tumor models. Further, all therapies were well tolerated without overt signs of toxicities and significant weight loss. Importantly, white cell and platelet counts, though reduced, stayed within normal ranges for all treatment groups (data not shown).

### Panobinostat/everolimus treatment inhibits cap-independent translation and not cap-dependent translation

**Figure 4:**
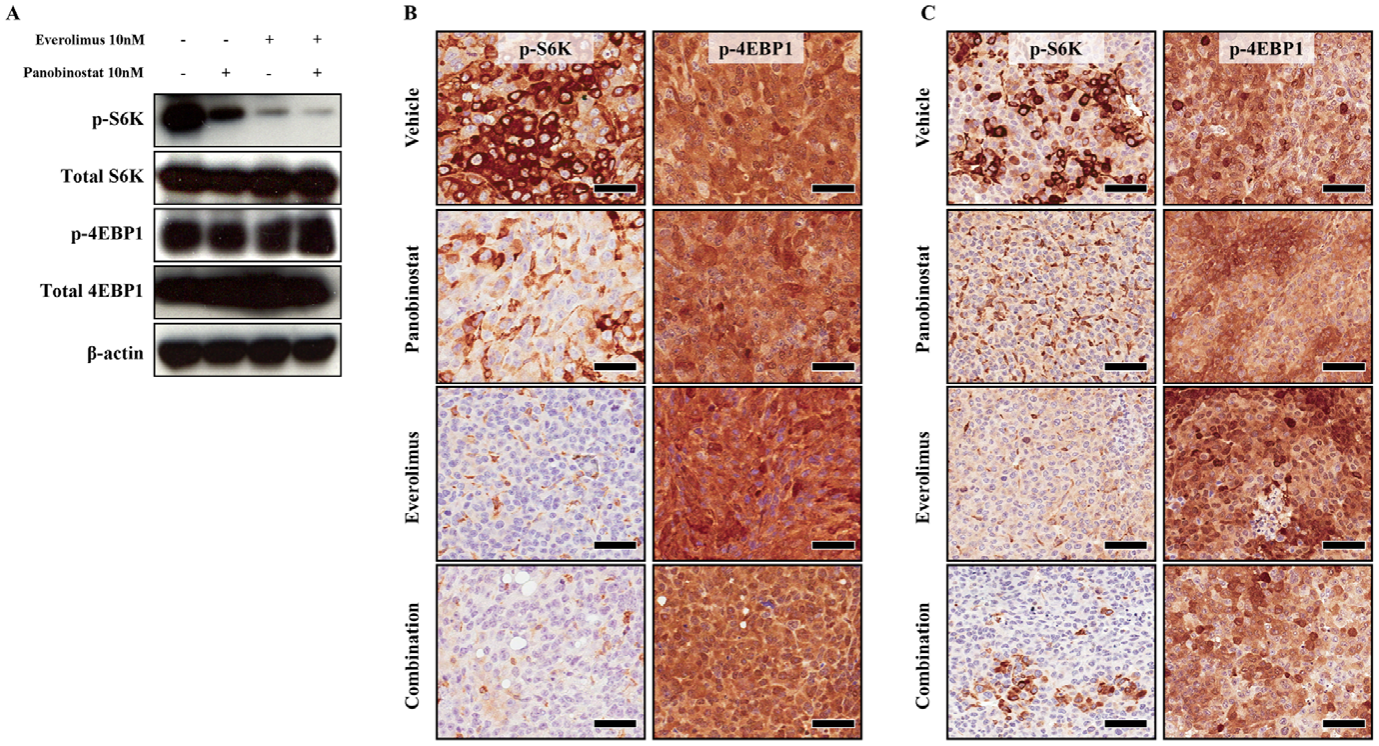
HDAC and mTORC1 inhibition attenuate cap-independent translation but not inhibit cap-dependent translation in Myc-CaP cell lines and tumors. (A) Western blot analysis of p-S6K, total S6K, p-4EBP1 and total 4EBP1 proteins. MycCaP cells were treated with 10 nM everolimus, 10 nM panobinostat or combination for 24 hours. Cell lysates were then subjected to immunoblot and incubated with the respective antibodies. β-actin served as loading control. (B) Myc-CaP/AS tumors or (C) Myc-CaP/CR tumors (∼5 mm^2^) were transplanted into the flank of wild type FVB intact male mice. Approximately ten (10) days post transplantation mice were treated daily with 10 mg/kg everolimus orally, 10 mg/kg panobinostat IP or both agents concurrently for a total of 15 days. Excised tumors were formalin-fixed paraffin and stained for the downstream targets of mTORC1 signaling, p-S6K and p-4EBP1. Magnification (×40); Scale bar = 50 µM.

It was recently demonstrated that over-expression of Myc resulted in incomplete loss of mTORC1 signaling by chemical inhibition [18]. We therefore wanted to determine if similar events were occurring within our model system with mTORC1 inhibition by everolimus. Myc-CaP cell lines treated with indicated concentrations of panobinostat, everolimus or combination for 24 hours and mTORC1 activity was evaluated by protein expression levels of phospho-S6K (p-S6K) and phospho-4EBP1 (p-4EBP1) by western blot. Figure 4A clearly indicates that single and combination treatment of Myc-CaP cells with panobinostat and everolimus inhibit cap-independent translation as indicated by loss of p-S6K, but does not result in inhibition of cap-dependent translation as indicated by p-4EBP1. Either single or combination treatment did not result in protein degradation as indicated by stable protein expression of unphosphorylated S6K and 4EBP1 (Fig. 4A). IHC staining was preformed on tumor tissue collected from described *in vivo* therapy experiments (Fig. 3) to confirm observed *in vitro* results. Both Myc-CaP/AS (Fig. 4B) and Myc-CaP/CR (Fig. 4C) tumors express abundant p-S6K and p-4EBP1 expression as indicated by vehicle treated tissue samples. Panobinostat and everolimus single treatments result in strong attenuation of p-S6K signaling in both androgen sensitive and castrate resistant tumors, while panobinostat/everolimus combination appears to have an additive effect of p-S6K signaling compared to single treatments. Signaling mediated by p-4EBP1 however in both androgen sensitive and castrate resistant tumors was not affected by panobinostat or everolimus single and combination treatments.

### Panobinostat/Everolimus combination attenuates Androgen Receptor and HIF-1α transcriptional activity *in vitro*

**Figure 5:**
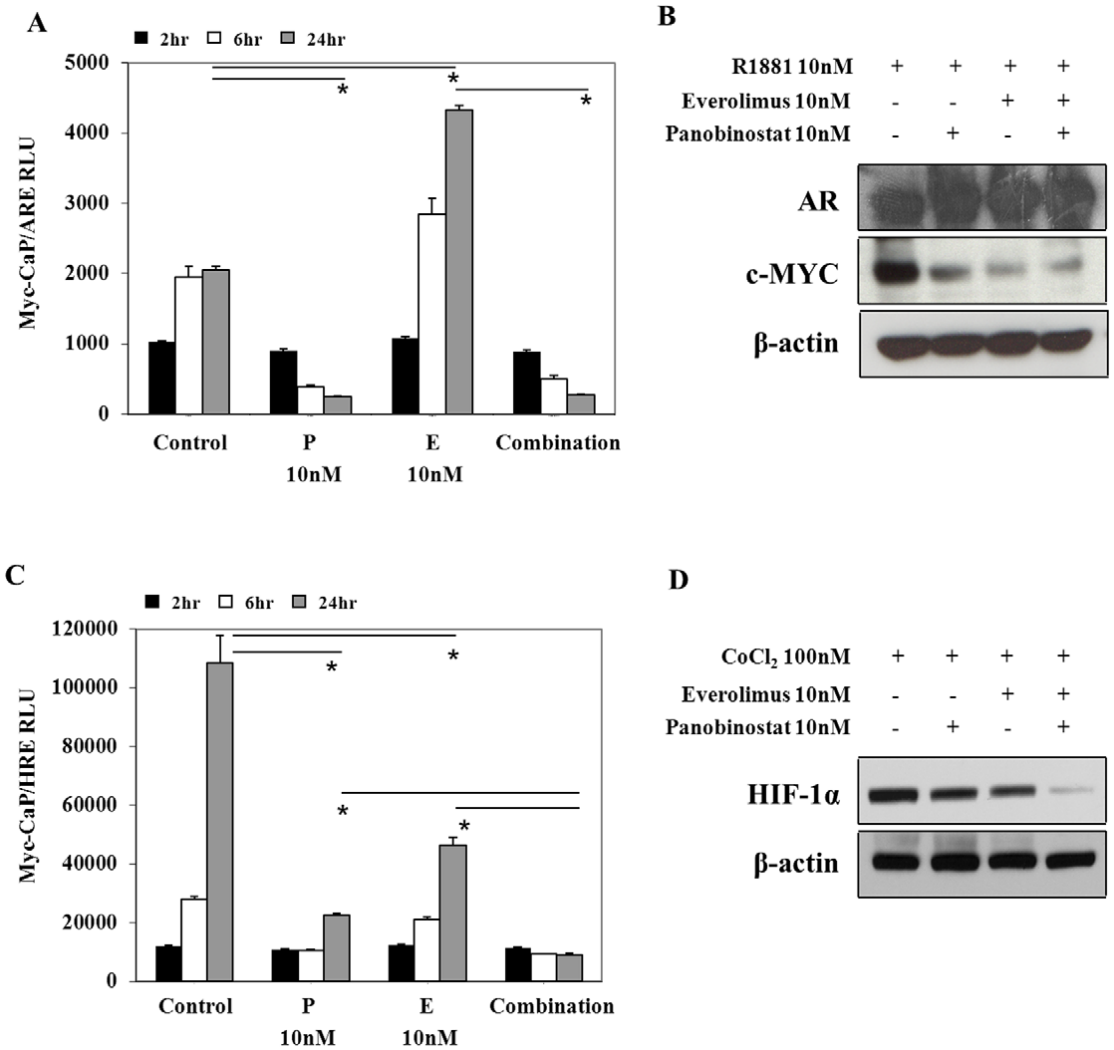
HDAC and mTORC1 inhibition in Myc-CaP cell lines *in vitro* result in alterations of androgen receptor and HIF-1α protein levels with loss of transcriptional activity. (A) Myc-CaP cell lines with stable expression of androgen response element [Myc-ARE] plasmid treated with 10 nM R1881±10 nM panobinostat [P], 10 nM everolimus [E] or combination. At indicated times cells were lysed and luminescence intensity was measured and quantitated. Mean ± SE from 3 independent experiments. (B) AR and human c-Myc protein was investigated by immunoblot using whole cell Myc-CaP lysates after treatment with 10 nM R1881 with or without 10 nM panobinostat, 10 nM everolimus or combination for 24 hours. β-actin was used as loading control. (C) Myc-CaP cell lines with stable expression of hypoxia response element [Myc-HRE] plasmid treated with 100 µM CoCl_2_±10 nM panobinostat [P], 10 nM everolimus [E] or combination. At indicated times cells were lysed and luminescence intensity was measured and quantitated. Mean ± SE from 3 independent experiments. (D) HIF-1α protein was investigated by immunoblot using whole cell Myc-CaP lysates treated with 100 µM CoCl_2_ with or without 10 nM panobinostat, 10 nM everolimus or combination for 24 hours. β-actin was used as loading control. * p<0.05.

Transcriptional activity of AR and HIF-1α are considered important for PCa growth and survival. Our laboratory had previously demonstrated that the combination of rapamycin and panobinostat resulted in HIF-1α protein degradation associated with a reduction in tumor angiogenesis of prostate and renal cell carcinoma xenograft models [19]. Further, HDACI have the ability to disrupt AR protein stability and transcription [14] and there has been recent work investigating mTOR and AR cross talk [20]. Myc-CaP/ARE cells cultured in low androgen conditions supplemented with 10 nM R1881 demonstrate that ligand dependent AR transcriptional activity is inhibited by panobinostat, whereas everolimus treatment resulted in a significant increase in AR transcriptional response. Combination treatment showed that panobinostat was able to significantly inhibit the activation of AR transcriptional response mediated by everolimus (Fig. 5A). Investigation of whole cell Myc-CaP lysates by immunoblot indicate that either single or combination treatment does not result in AR protein degradation, but rather an increase in AR protein levels compared to R1881 alone treated cells. As indicated by decreased proteins levels of c-Myc (specific transcriptional target of AR in this model), AR transcriptional activity is suppressed (Fig. 5B). It is possible that the opposing effects on luciferase and Myc expression by AR transcriptional activity in Fig. 5A and Fig. 5B by treatment with everolimus maybe explained through the ability of everolimus alone or in combination to only inhibit cap-independent (dependent for c-Myc translation) and not cap-dependent translation (dependent for luciferase translation) (Fig. 4).

The transcriptional activity of HIF-1α was assessed by the use of reporter plasmids expressing hypoxia response element specific for the recognition by HIF-1α and not HIF-2α. Myc-CaP/HRE cells treated *in vitro* with cobalt chloride to mimic hypoxia and demonstrate a HIF-1α time dependent response that is significantly inhibited by panobinostat and everolimus single treatments, though combination of these two drugs produced a significant reduction of HIF-1α transcriptional activity compared to single treatments (Fig. 5C). Further, western blots performed indicate that panobinostat/everolimus combination greatly reduced HIF-1α protein levels compared to single treatments (Fig. 5D).

### Panobinostat/everolimus combination attenuates Androgen Receptor and HIF-1α transcriptional activity *in vivo*

**Figure 6:**
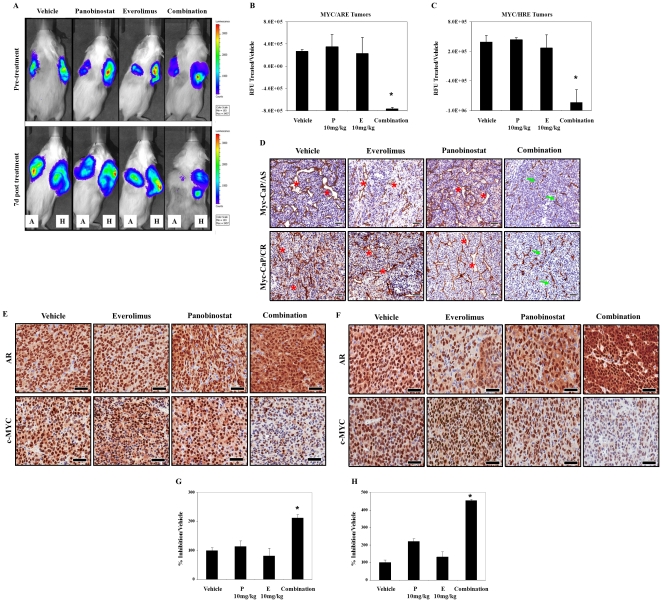
HDAC and mTORC1 inhibition in combination mediate greatest increased loss in Myc-CaP tumor angiogenesis and androgen receptor and HIF-1α transcriptional activity *in vivo*. (A) Myc-CaP tumors expressing either ARE [A] or HRE [H] plasmids were transplanted bi-laterally to wild type FVB intact male mice. Mice were treated daily with 10 mg/kg everolimus orally, 10 mg/kg panobinostat IP or combination for a total of 7 days. (B–C) Luminescence was measured and quantitated pre- and post therapy. Mean ± SE, *n* = 3. (D) Tumor samples from figure 3A and 3E were collected and placed in zinc fixative. Four micron (4 µM) sections were stained for the endothelial marker CD31. Magnification (×20); Scale bars = 50 µM. (E–F) Tumor samples from figure 3A and 3E were collected and fixed in 10% neutral buffered formalin. Four micron (4 µM) sections were stained for AR and human c-Myc. Magnification (×40); Scale bars = 50 µM. (G–H) Quantitation of human c-MYC inhibition from Fig. 4E and F. Mean ± SE, *n* = 3 mice.

Because the transcriptional activity of AR and HIF-1α was attenuated by panobinostat/everolimus combination *in vitro*, we asked if these events were critical for the superior anti-tumor activity of panobinostat/combination therapy *in vivo*. Panobinostat/everolimus combination therapy significantly inhibits AR and HIF-1α transcriptional activity *in vivo* (Fig. 6A–C). Combination therapy also induced dramatic reduction of luminal structures throughout the tumor vasculature (*) as well as a dramatic reduction in the size of these vessels (indicated by arrows) compared to single treatment of Myc-CaP/AS and Myc-CaP/CR tumors (Fig. 6D). Further, single treatment of both tumors did not result in major changes of AR or c-Myc expression, whereas combination treatment strongly induced cytoplasmic localization of AR with an associated loss of c-Myc expression (Fig. 6E–H). These results indicate that combination anti-tumor activity may be in part mediated by the inhibition of angiogenesis by loss of HIF-1α activity and also via of AR transcriptional response resulting in loss of tumor proliferation.

### Panobinostat/everolimus combination reduces known onco-microRNA expression *in vivo*

**Figure 7:**
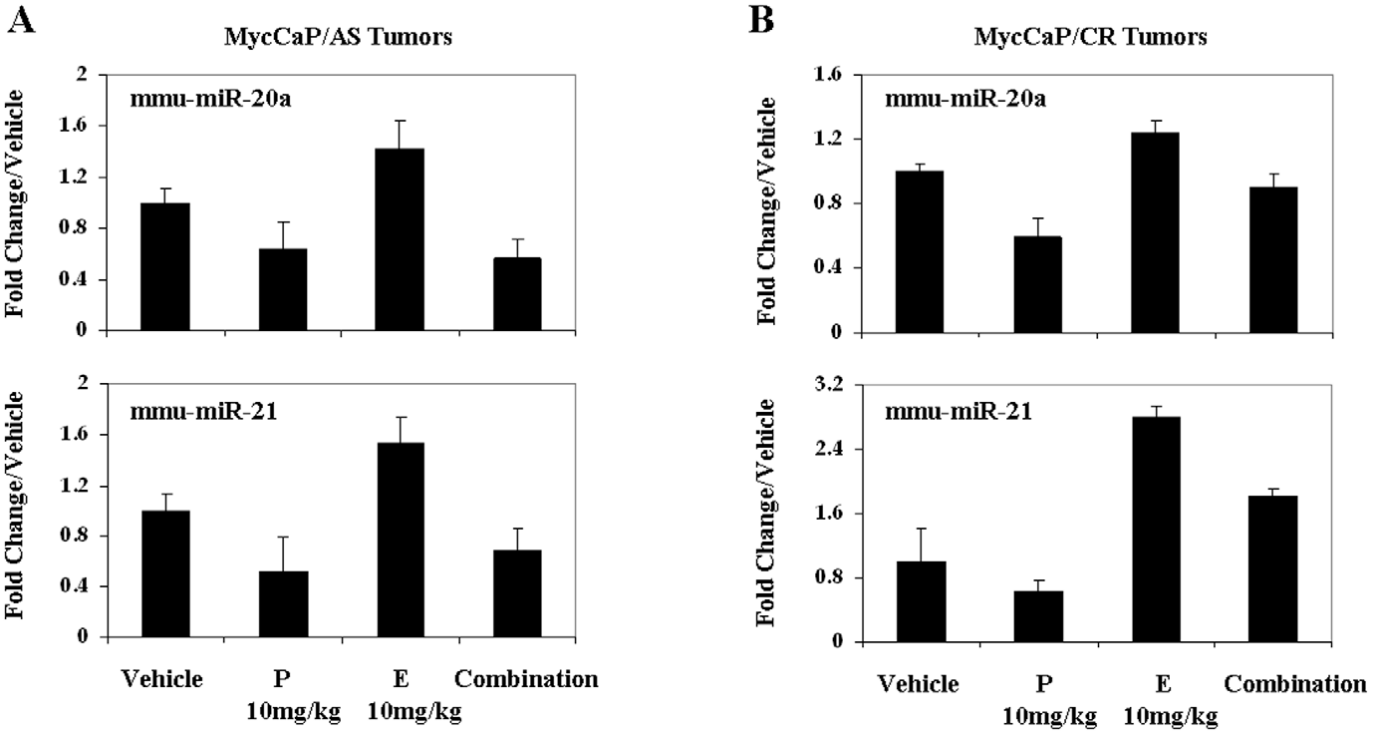
Inhibition of HDACs and mTORC1 *in vivo* differentially regulation expression of known oncogenic microRNA. RNA was extracted from (A) Intact mice bearing Myc-CaP/AS tumors and (B) castrated mice bearing Myc-CaP/CR tumors that were treated daily with 10 mg/kg everolimus (E) orally, 10 mg/kg panobinostat (P) IP or combination for a total of 7 days. QRT-PCR analysis was used to generate ΔΔCt values to investigate microRNA expression, which is expressed as fold change over control samples. *n = 2*. Mean ± SE of ΔΔCt values from 2 independent tumors.

**Figure 8:**
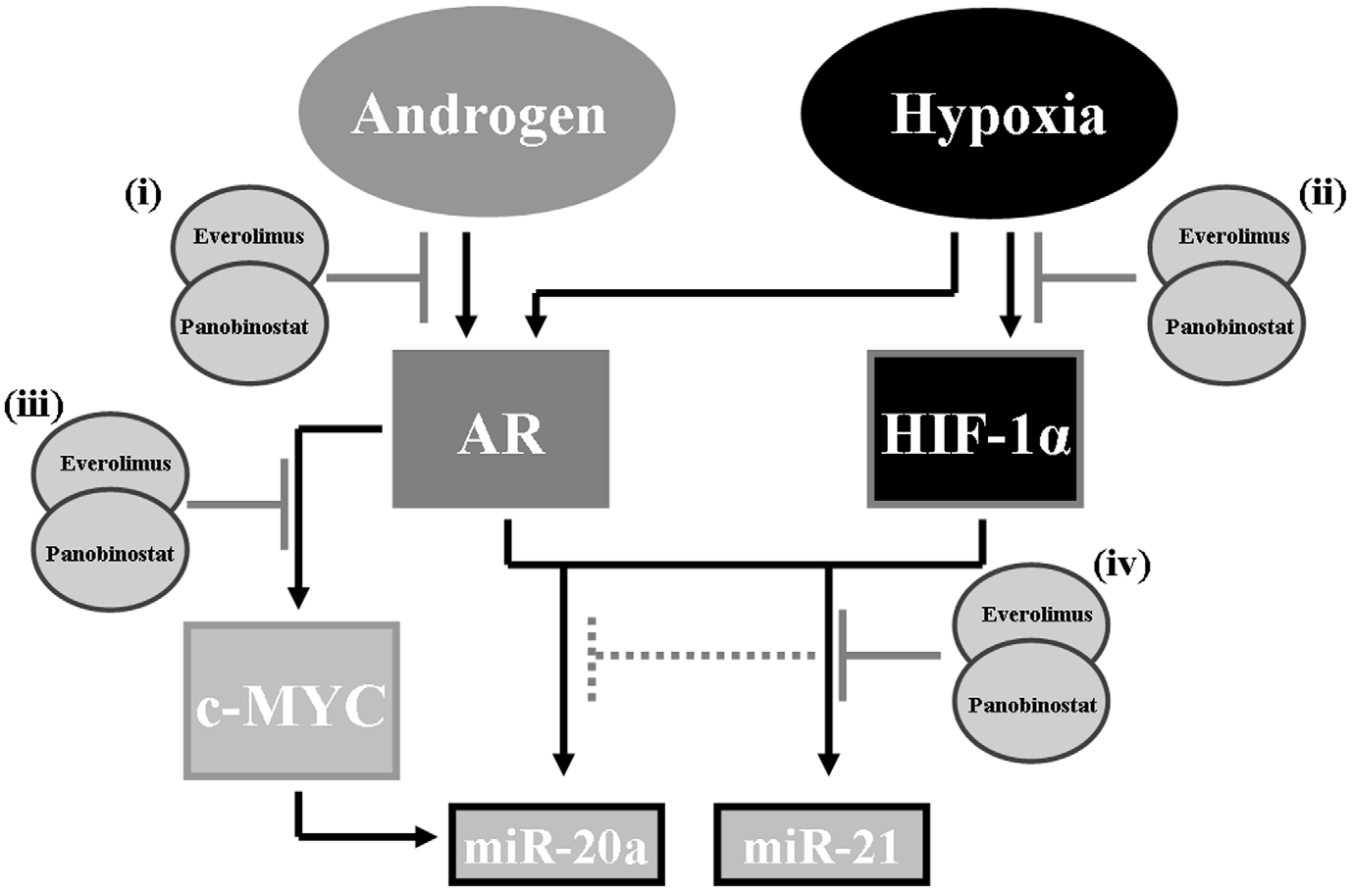
HDAC and mTORC1 inhibition antagonize androgen and hypoxia mediated signaling. Schematic diagram illustrating potential points where combination therapy attenuates hypoxia and androgen signaling in murine Myc-CaP PCa tumors. (i) Upstream of ligand mediated AR activity, (ii) Upstream of hypoxia mediated HIF-1α activity, (iii) Downstream of AR transcriptional activity and (iv) Downstream of HIF-1α transcriptional activity.

Hypoxia, AR and c-Myc signaling have been documented to target downstream microRNA's via their transcriptional activity. Because our previous results demonstrate decreased oncogene signaling via attenuation of HIF-1α and AR (also c-Myc) transcriptional activity we investigated known associated onco-miRs downstream of these transcription factors that may indicate potential mechanisms of panobinostat/everolimus combination anti-tumor activity. Using QRT-PCR, we determined the expression levels of a documented miR associated with AR/hypoxia signaling, miR-21 [21] and the c-Myc/hypoxia associated miR-20a [22]. Regulation of miR expression patterns in both Myc-CaP/AS and Myc-CaP/CR by panobinostat single treatment resulted in down-regulation of miR-20a and miR-21 compared to vehicle treated mice. Response to everolimus single treatment however resulted in both miRs being up-regulated respective to control treated mice. The up-regulation of these two onco-miRs was attenuated in the panobinostat/everolimus combination treated mice (Fig. 7A and Fig. 7B). Taken together these data demonstrate that inhibition of HDACs and mTORC1 can affect androgen and hypoxia signaling at multiple levels (Fig. 8). By combining everolimus with panobinostat we elude possible tumor escape mechanisms in response to mTOR inhibition (increased AR transcriptional activity and expression of known onco-miRs), resulting in, at least with this combination, cytostatic anti-tumor activity.

## Discussion

HDAC inhibitors exhibit pleiotropic molecular and biologic effects [12], [23] and have shown clinical activity in the treatment of cutaneous T cell lymphoma [24]. Because of HDAC inhibitors ability to affect multiple pathways and genes involved in apoptosis [25], cell cycle arrest [26] and angiogenesis [24], [27], [28], their greatest potential as targeted therapies maybe to be utilized in novel combinational therapeutic strategies in PCa with already existing chemotherapies such as docetaxel [29], or with other novel targeted chemotherapies including mTOR inhibitors [19].

Within, we have utilized the mouse cell line Myc-CaP [16] generated from the Hi-Myc transgenic mouse model of prostate cancer [17], which drives the expression of human c-Myc by the androgen receptor dependent rat probasin promoter, to assess the *in vitro* and *in vivo* anti-tumor activity of combination treatment with low dose HDACI panobinostat and the mTOR inhibitor everolimus. It has been documented that PCa involves deregulated expression of HDACs [6] and activation of mTORC1 [30], [31] signaling and therefore provide rationale to target these in combinational therapeutic strategies.

Initial *in vitro* data demonstrates that low dose panobinostat/everolimus combination did not result in tumor cell apoptosis, but rather reduced the tumor growth and clonogenic capacity of Myc-CaP cell lines through induction of cell cycle arrest associated with enhanced p21 and p27 protein expression. This is consistent with recent data published that demonstrated HDAC/mTOR inhibitor combination treatment of PCa cell lines resulted in increased inhibition of cell growth and cell cycle progression concurrent with increased levels of p27 and p21 proteins [32].. Previous studies indicate that inhibition of HDAC inhibitor mediated apoptosis is abrogated through induction of p21 and/or p27. Inhibition of p21 and/or p27 by chemical or molecular techniques sensitized cancer cell lines to the apoptotic inducing abilities of HDAC inhibitors [33], [34]. Also, inhibition of mTORC1 often results in feedback activation of Akt by mTORC2 [8]. Activated Akt can also inhibit apoptosis via phosphorylation of critical pro-apoptotic proteins including Bim and Bad [35] adding possible mechanistic explanation as to why panobinostat/everolimus combination induces tumor-static rather than tumor-cidal effects within this model. Moreover, p21 repression is mediated by c-Myc and induced acetylation of the p21 promoter through HDAC inhibition and loss of c-Myc expression is correlated with induced p21 expression [36]. Likewise, our treatment of Myc-CaP cell lines with panobinstat did induce histone H3 acetylation (data not shown) and combination with everolimus resulted in greatest inhibition of c-Myc protein expression. *In vitro* growth inhibition by panobinostat/everolimus combination was also correlated *in vivo* in our syngeneic mouse transplant model, where Myc-CaP/AS and Myc-CaP/CR tumor growth was inhibited without induction of tumor apoptosis as determined by caspase 3 staining (data not shown). Reduction in tumor burden has been previously demonstrated in a PCa xenograft model treated with concurrent HDAC/mTOR inhibition [19] and more recently in renal cell carcinoma (RCC) xenograft models [37]. HDAC/mTOR inhibition in these RCC models also resulted in tumor apoptosis via greater inhibition of the anti-apoptotic, pro-angiogenic protein survivin [37], [38]. Survivin was also expressed in Myc-CaP/AS tumors and similar attenuation of survivin expression also occurred in response to panobinostat/everolimus combination therapy (Fig. S1). Even with significant loss of survivin expression, tumor apoptosis was not induced as demonstrated by Mahalingam et al. This indicates that Myc-CaP tumor blockade of apoptosis is independent of survivin. Both cap-dependent and cap-independent pathways are usually strongly attenuated following mTORC1 inhibition [39]. Further, it has also been demonstrated that enforced expression of *c-Myc* can abrogate sensitivity to mTORC1 inhibition by the rescue of the 4EBP1 mediated cap-dependent translation signaling pathway [18]. Our *in vitro* and *in vivo* data also show that within our Myc driven model that cap-dependent translation is not inhibited by loss of mTORC1 activity though c-Myc expression is attenuated, which suggests that rescue of cap-dependent translation can also be independent of c-Myc. The resistance of cap-dependent translation to mTORC1 inhibition observed here may also provide alternate escape mechanisms as cap-dependent translation is responsible for the expression of well known onco-genes including *cyclin D1* [40].

Alternatively, HDAC inhibitors of the same class including vorinostat and panobinostat elicit overlapping gene transcription patterns, but they can also mediate specific genetic signatures, possibly because of different HDAC inhibition capabilities [12], [41]. Hierarchical clustering of expression profiles from 3 independent cancer cell lines treated with they hydroxamic acid HDACI vorinostat and TSA regulated 8–10% of genes which included only a core set of 13 (1–2% of the total genes regulated by HDACI) genes which were regulated similarly by both hydroxamic acid HDACI [42]. Further, it was also demonstrated that panobinostat possesses a greater affinity for binding all HDAC isoforms when compared to other hydroxamic acid HDACI including vorinostat and belinostat [41]. These data highlight the possible importance of understanding HDAC expression underlying specific tumor types which may aide in HDAC inhibitor type and dose used to treat PCa patients.

HDAC and mTOR inhibitors also demonstrate greater anti-angiogenic activity in combination [19], [37]. Recent data published from our laboratory, displays combination of rapamycin and panobinostat significantly reduced HIF-1α protein and vessel density in xenograft models with constitutive mTOR activity, either through loss of *Pten* (PC3 cells) or *VHL* (C2 cells) [19]. Myc-CaP/AS and Myc-CaP/CR tumors express wild type Pten and low levels of activated mTOR (data not shown). Even so, we observed substantial activity in HIF-1α transcriptional activity associated with densely vascularized tumors. Panobinostat/everolimus combination resulted in abundant inhibition of tumor angiogenesis in androgen sensitive and castrate resistant tumors. We believe that the highly vasculature phenotype in Myc-CaP tumors is driven by c-Myc expression itself, as c-Myc has been shown to be essential for vasculogenesis and angiogenesis during tumor development and progression [43]. Further, increased proliferation of c-Myc driven tumors creates a greater environment of tumor hypoxia which in turn activates HIF-1α activity. Also, enhanced metabolic stress within the tumor cell could allow for mTORC1 inhibition to elicit a therapeutic response in combination with HDAC inhibitors.

Critical to androgen sensitive and castrate resistant prostate cancer growth and survival is the transcriptional activity of the AR. Myc-CaP cell lines express an amplification of their wild type AR though this phenomenon was independent of androgen withdrawal [16]. HDAC inhibitors have previously demonstrated the ability to attenuate AR transcriptional activity by either loss of protein expression or by disabling the ability of AR to bind DNA [14], [44]. Conversely, inhibition of mTORC1 signaling activates AR signaling [20], [45]. Our *in vitro* analysis indicates in line with previous reports that AR transcriptional activity is increased following mTORC1 inhibition and combination with a HDACI attenuates AR transcription mediated by loss of mTORC1. Surprisingly, HDACI mediated inhibition of AR transcription was not a result of loss of AR protein expression. HDAC and mTORC1 inhibition increased AR protein expression. This observation also occurred within other investigations including those by Iacopino *et al.* who showed that treatment of LNCaP and PC-3 cells *in vitro* for 4 days with valproic acid resulted in increased AR protein levels over control treated cells [44]. Further, Schayowitz *et al.* demonstrated that *in vivo* treatment of HP-LNCaP tumors with everolimus resulted in increased AR protein levels compared to control treated tumors [46]. Also, Welsbie *et al.* demonstrated that HDACI treatment of LNCaP cells resulted in decreased AR protein and mRNA 24 hours post treatments. They concluded in further experiments that the dominant reason for loss of AR protein levels was a result of transcriptional repression of *AR* and not enhanced protein degradation following HDAC inhibition [14]. Experimental differences may explain the discrepancies between HDACI mediated effects on AR expression, where Welsbie *et al.* used normal culture conditions to explore effects on AR expression by HDACI. Our data presented within were conducted in androgen supplemented culture conditions like Iacopino *et al.* which would result in increased AR protein stability. Later time points like those by Iacopino *et al.* may have resulted in loss of AR protein expression in our model system. Overall, our data indicates elevated AR transcriptional activity resulting from mTORC1 inhibition was significantly inhibited by panobinostat in combination treatment, indicating increased AR function can maintain survival in the presence of mTORC1 inhibition, and to perturb AR function with HDAC inhibitors offers a novel therapeutic strategy to over come this.

Recently, microRNAs importance as effectors of hypoxia, c-Myc and AR signaling has been recently highlighted [47], [48], [49], [50]. Of specific interest to us was the response of two documented microRNAs to exhibit oncogenic activity in PCa and whose expression is mediated by these signaling pathways, miR-20a [51] and miR-21 [52]. Recently, miR-21 was observed to be elevated in patient serum levels with metastatic hormone-refractory PCa [53]. Further, when serum miR-21 and miR-141 levels was integrated with PSA serum levels, positive prediction of PCa was increased from 40% to 87.5% success [54]. Also, data from clinical samples indicated that patients with a Gleason score ≥7 had significant increase of miR-20a expression compared to patients with Gleason score ≤6 [51]. Treatment of Myc-CaP/AS and Myc-CaP/CR tumors resulted in similar responses where both onco-miRs were up-regulated in response to everolimus treatment, though excitingly panobinostat treatment attenuated this increase in onco-miR expression. The down regulation of these two associated PCa microRNAs raises the opportunity to possibly evaluate patient response to therapy and to predict the efficacy of these targeted therapies on important signaling pathways involved in PCa. Whereas PSA allows for surveillance of AR transcriptional activity, microRNAs including miR-20a and miR-21 would allow the monitoring of multiple pathways within PCa patients being treated with novel targeted therapies.

Androgen receptor, c-Myc and HIF-1α activity are associated with poor prognosis in many cancers, including PCa [55], [56]. Previous work from this laboratory has demonstrated panobinostat to be potent inhibitor of tumor angiogenesis as a single agent [27] and also in combination with the mTORC1 inhibitor, rapamycin [19]. These studies were conducted in the PC3 PCa tumor model which has constitutive activation of the PI3K-Akt-mTOR pathway through loss of Pten. Those data focus on the mediation of antitumor activity by panobinostat's ability to induce HIF-1α protein degradation in endothelial cells, thus inhibiting tumor angiogenesis and potentiating anti-tumor activity. Our current investigation utilizes an immunocompetent syngeneic mouse model of PCa which is Pten expressing and does not involve constitutively activated PI3K-Akt-mTOR signaling. Collectively, our data presented within demonstrate that only panobinostat/everolimus combination therapy result in the degradation of HIF-1α protein and inhibits HIF-1α and AR transcriptional activity *in vivo*. The low dose biological effects of this combination are of particular interest in view of a previous report showing poor tolerability and limited activity of full dose of vorinostat in patients with advanced CRPC [57]. Also, to date single agent clinical activity of either HDAC or mTOR inhibition in PCa has been limited [57], [58]. This study provides strong rationale for the continued clinical investigation and design of clinical trials with rational combinations of targeted therapies including HDAC and mTOR blockade for the treatment of patients with advanced and castrate resistant PCa.

## Materials and Methods

### Ethics Statement

The Institute Animal Care and Use Committee (IACUC) at Roswell Park Cancer Institute (RPCI) approved all mouse protocols used in this study. Our approval/protocol ID is 1137M.

### Cell culture and reagents

The Myc-CaP cell line [16] was a kind gift from Dr Charles Sawyers and were cultured in DMEM medium (Gibco) supplemented with 10% fetal bovine serum and 1% penicillin/streptomycin at 37°C, 5% CO_2_. *In vitro*, panobinostat and everolimus powder (Novartis) were dissolved in DMSO as 10 mM stocks and diluted in cell culture medium prior to experiments. *In vivo*, panobinostat powder was dissolved in D5W (5% dextrose in distilled water) at a concentration of 1 mg/mL. Everolimus was supplied as an aqueous solution at 20 mg/mL and diluted in distilled water to a final concentration of 1 mg/mL. A placebo (vehicle) was also supplied as an aqueous solution and diluted in distilled water the same as everolimus. Cobalt chloride was purchased from Sigma-Aldrich. Lentiviral particles containing reporter element constructs for androgen receptor (AR) (product CLS-8019L) and hypoxia inducible factor-1 alpha (HIF-1α) (product # CLS-007L) response elements, which drive firefly luciferase expression, where purchased from SABiosciences. Bright-Glo™ Luciferase Assay System (Promega) was used to detect luciferase luminescence for *in vitro* assays. Antibodies used for western blot or IHC staining were AR (Santa Cruz Biotechnology), c-Myc (Epitomics), total and phospho-S6K (Cell Signaling), total and phospho-4EBP1 (Cell Signaling), p21 (Santa Cruz Biotechnology), p27 (Cell Signaling) anti-HIF-1α (Cayman Chemicals), CD31 (BD Pharmingen) survivin (Santa Cruz Biotechnology) and β-actin (Sigma-Aldrich).

### In vitro cell death and cell growth assays

Myc-CaP cells (4×10^5^/mL) were left to adhere overnight in 24-well plates (BD Biosciences) then incubated in the presence of indicated treatments for 24–48 hours in 1 mL normal cell culture medium. Viability (cell death) was measured by propidium iodide (Sigma) (PI) uptake. Apoptosis was measured by annexin V (BD Bioscience) and PI double staining. Cell growth was measured by fixation and staining of cells with 10% Methanol/Crystal Violet solution. Stained cells were made soluble in absolute methanol and absorbance was detected at an emission length of 570 nm.

### Clonogenic survival assays

Myc-CaP cells (5×10^2^/mL) were left to adhere overnight in 6-well plates. Cells were then treated as indicated for 24–48 hours. Post drug treatment cells were washed in fresh media and grown in the absence of drug for 12 days. Developed cell colonies were fixed and stained in 10% Methanol in Crystal violet solution. Colony counts were performed using Image J software.

### Western blot analysis

Myc-CaP were washed in PBS and lysed in RIPA buffer (Sigma-Aldrich) containing 1× protease and phosphatase inhibitors (Sigma-Aldrich). Equal amounts of protein were separated by electrophoresis using 4–15% SDS-PAGE gradient gels (Bio-rad) as previously described [25]. Protein was transferred to nitrocellulose membranes.. Anti-rabbit and mouse horseradish peroxidase-conjugated secondary antibodies were from Dako (Carpinteria, CA). Immunoblots were visualized using enhanced chemiluminescence (PerkinElmer).

### Cell Cycle Analysis

Myc-CaP cells (4×10^5^/mL) were left to adhere overnight in 6-well plates. Cells were then treated with indicated compounds for 24 and 48 hours. Adherent and non-adherent cells were collected and washed in PBS. Cells were fixed over night in 50% ethanol and stained with PI solution containing RNase A (Sigma) for 15 minutes at 37°C. DNA content was analyzed using a FACS Caliber cytometer.

### *In vitro* analysis of AR and HIF-1α transcriptional activity

To generate Myc-CaP cell lines stably expressing ARE/luciferase (Androgen Response Elements; Myc/ARE) or HRE/luciferase (HIF-1α Response Elements; Myc/HRE), Myc-CaP cells were grown to 70% confluency in a 96 well plates (BD Biosciences) and transduced with lentiviral particles containing ARE/luciferase or HRE/luciferase expression plasmids according to manufactures instructions (SABiosciences). Stably expressing cells were selected by resistance to puromycin (2 µg/mL) over 14 days. Luminescence quantitation was measured from Myc-CaP cell lines by Bright-Glo™ Luciferase Assay System.

### RNA extraction and Quantitative real-time PCR

RNA was extracted from treated and untreated Myc-CaP/AS and Myc-CaP/CR tumors by TRI Reagent® (TRIzol) method. For first strand cDNA synthesis 40 ng of total RNA was reverse transcribed into a final volume of 20 ul using the miRCURY LNA™ Universal RT cDNA synthesis kit (Exiqon) as per manufacturer's instructions. One microliter of synthetic spike-in was added to 40 ng of FirstChoice Human Placental Total RNA (Ambion) and reverse transcribed. This sample was run as an inter-plate calibrator on every plate using control primers supplied with the Exiqon SYBR green master mix. This control allows for the detection of run-to-run variation between plates. Upon completion of the RT process, the template is used for real-time PCR amplification. For QRT-PCR amplification, the cDNA template is diluted 80-fold in nuclease-free water prior to use. Ten Microliter reactions were carried out, in triplicate, according to manufacturer's specifications (Exiqon) using SYBR Green master mix (Exiqon) and pre-designed LNA PCR primer sets (listed below). 40 cycles of PCR amplification, (initial 95°C denaturation step for 10 minutes followed by 40 cycles of 95 C for10 seconds then 60°C for one minute) were performed using relative quantitation on a 7900HT Sequence Detection System (ABI) with optical reading of the SYBR green signal during the 60°C annealing/extension step. Data analysis was performed using the ABI 7900HT SDS software v2.4 and RQ manager 1.2.1. The microRNA target sequences are miR21 (UAGCUUAUCAGACUGAUGUUGA) which produces a product of 44 base pairs and miR20a (UAAAGUGCUUAUAGUGCAGGUAG) which produces a product of 47 base pairs. U6 was used has a control reference gene. Product numbers for used primers were 204230 for miR21 and 204292 for miR20a. For proprietary reasons the Exiqon oligonucleotide (primer) sequences are unable to be published. ΔΔCt values were calculated for miR for two separate mice and used to express mRNA as fold change of treated over control.

### *In vivo* mouse experiments

For *in vivo* therapy experiments the generation of Myc-CaP tumor banks was first established. These tumor banks consisted of Myc-CaP androgen sensitive tumors (Myc-CaP/AS), Myc-CaP castrate resistant tumors (Myc-CaP/CR) (described in [59]), Myc-CaP/AS-ARE and Myc-CaP/AS-HRE (both Myc-CaP/ARE and Myc-CaP/HRE tumors are androgen sensitive). All mice were purchased from NCI Frederick (Maryland, USA).

*Development of Myc-CaP tumor banks*: Myc-CaP cells (1×10^6^ cells/mouse in 200 µL volume of PBS) were injected subcutaneous into wild-type FVB male mice. Resulting Myc-CaP/AS and Myc-CaP/CR tumors were resected and viable tumor tissue was stored −80°C until use. Established Myc-CaP/AS-ARE and Myc-CaP/AS-HRE tumors were confirmed by bioluminescence imaging using the Xenogen® IVIS 50 system. Tumors positive for ARE or HRE driven luciferase expression were resected and viable tumor tissue was stored at −80°C until use.

*In vivo therapy experiments with mice bearing Myc-CaP/AS and CR tumors*: Intact or castrated male FVB mice received small pieces of Myc-CaP/AS or Myc-CaP/CR tumor tissue (∼5 mm^2^) respectively by subcutaneous implantation. Tumor growth was monitored by caliper measurement. Ten (10) days post engraftment mice received treatment with everolimus by oral gavage (10 mg/kg) daily, panobinostat (10 mg/kg) by intraperitoneal (IP) injections daily, or both therapies in combination daily. Mice in the control group received a corresponding amount of placebo administered by oral gavage. Anti-tumor activity was determined by serial caliper measurements and all tumor tissue collected post-mortem was weighed and used in immunohistochemical studies. Blood was collected by retro-orbital methods at the experiments conclusion to investigate peripheral white cell and platelet counts.

*In vivo therapy experiments with mice bearing Myc-CaP/ARE and Myc-CaP/HRE tumors*: Intact male FVB mice received small pieces of Myc-CaP/ARE and Myc-CaP/HRE tumor tissue (∼5 mm^2^) bilateral by subcutaneous implantation. Mice were treated as described above for a total of 7 days. *In vivo* imaging to determine tumor androgen receptor and HIF-1α transcriptional activity was carried out by Myc-CaP/ARE and Myc-CaP/HRE tumor bearing mice being anesthetized using isoflorane and bioluminescence imaging was conducted using a Xenogen® IVIS 50 system.

### Immunohistochemistry

Formalin fixed, paraffin-embedded tissue (4 µm) were stained with primary antibodies. All sections were incubated overnight with primary antibodies or respective IgG controls at 4°C and then incubated with ImmPRESS™ reagent kit HRP anti-rabbit IgG antibodies (Vector Laboratories). Staining was developed by incubation with 3,3′-diaminobenzide (Dako), and counterstained with hematoxylin. Images were captured using a Scanscope XT system (Aperio Imaging) and analyzed using Imagescope software (Aperio).

### Statistics analysis

Statistical significance between treatment groups was determined using a Student's *t* test. Differences at P<0.05 were considered significant.

## Supporting Information

Figure S1 **HDAC in combination with mTORC1 inhibition attenuate expression of survivin** ***in vivo*** **.** Tumor cell lysates were created from excised Myc-CaP/AS tumor tissue (Fig. 3A–D) after being treated daily with 10 mg/kg everolimus orally, 10 mg/kg panobinostat IP or both agents concurrently for a total of 15 days. Cell lysates were then subjected to western blot incubated with the respective antibodies. β-actin served as loading control.
(TIF)
