# Supplementary figures and images for: Concurrent HDAC and mTORC1 Inhibition Attenuate Androgen Receptor and Hypoxia Signaling Associated with Alterations in MicroRNA Expression
Source: PLoS One. 2011 Nov 7;6(11):e27178. doi: 10.1371/journal.pone.0027178 (PMC3210144; doi:10.1371/journal.pone.0027178)

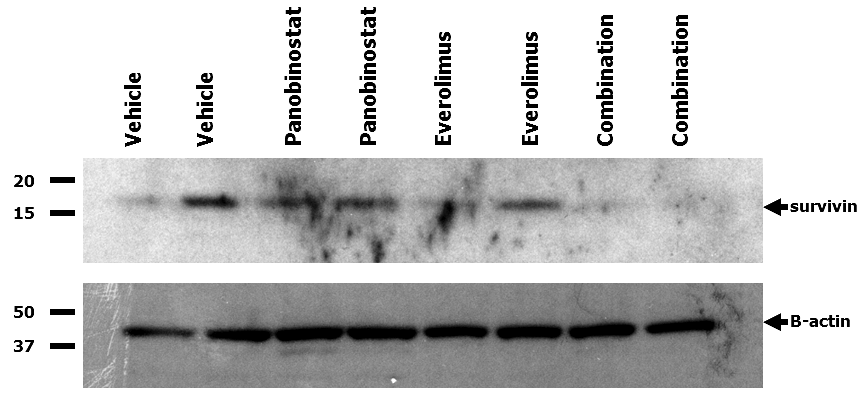

Supplement: Figure S1 — HDAC in combination with mTORC1 inhibition attenuate expression of survivin in vivo . Tumor cell lysates were created from excised Myc-CaP/AS tumor tissue (Fig. 3A–D) after being treated daily with 10 mg/kg everolimus orally, 10 mg/kg panobinostat IP or both agents concurrently for a total of 15 days. Cell lysates were then subjected to western blot incubated with the respective antibodies. β-actin served as loading control. (TIF) [file pone.0027178.s001.tif]
